# Superamphiphobic Cu/CuO Micropillar Arrays with High Repellency Towards Liquids of Extremely High Viscosity and Low Surface Tension

**DOI:** 10.1038/s41598-018-37368-y

**Published:** 2019-01-24

**Authors:** Qing Zhu, Bucheng Li, Shangbin Li, Guan Luo, Baohui Zheng, Junping Zhang

**Affiliations:** 1 Institute of Chemical Materials, China Academy of Engineering Physics, 621999 Mianyang, P. R. China; 2 Key Laboratory of Clay Mineral Applied Research of Gansu Province, Lanzhou Institute of Chemical Physics, Chinese Academy of Sciences, 730000 Lanzhou, P. R. China

## Abstract

For almost all the research of super anti-wetting surfaces, pure liquids like water and *n*-hexadecane are used as the probes. However, liquids of diverse compositions are used in academic research, industrial production and our daily life. Obviously, the liquid repellency of super anti-wetting coatings is highly dependent on properties of the liquids. Here, we report the first superamphiphobic surface with high repellency towards liquids of extremely high viscosity and low surface tension. The surfaces were prepared by constructing a hierarchical micro-/nanostructure on the Cu micropillar arrays followed by modification with perfluorosilane. The surfaces are superamphiphobic towards the liquids with extremely high viscosity and low surface tension because of (i) the micro-/nanostructured surface composed of micropillars with proper pillar distance and CuO nano-flowers, and (ii) the abundant perfluorodecyl groups on the surface. The contact angles, sliding angles, apparent contact line at the solid-liquid interface and adhesion forces are the end products of micropillar distance, viscosity and surface tension. Smaller micropillar distance, higher viscosity and higher surface tension contribute to reducing the adhesion force. We *in situ* observed the process of microcapillary bridge rupture for the first time using highly viscous liquids. We also successfully reduced the adhesion forces and enhanced the average rolling velocity of liquids with extremely high viscosity and low surface tension by regulating the micropillar distance.

## Introduction

Inspired by the unique wettability of the lotus leaf^1,2^, artificial super anti-wetting surfaces such as superhydrophobic^3–5^ and superamphiphobic surfaces^6–8^ have generated great attention in academia and industry. Super anti-wetting surfaces have wide potential applications in many fields including self-cleaning surfaces^9^, oil/water separation^10,11^, anti-corrosion^12,13^, anti-icing^14,15^ and anti-wetting textiles^16,17^. Thus, a wide range of methods and materials have been used to prepare super anti-wetting surfaces^18–23^. More specifically, to understand the solid-liquid interaction (e.g., impinging/bouncing^24–26^, sliding^27^ and modeling/simulation^28,29^) and to move the surfaces to real world applications (e.g., enhancing mechanical stability^30–32^, enhancing transparency^33,34^, waterborne^35,36^, flexible^37,38^ and applications^39–42^) are now in the spotlights in the field of super anti-wetting surfaces.

For all the fundamental and applied research of super anti-wetting surfaces, the repellency of the surfaces to liquids is crucial. While most of the attention has been being paid to the surfaces, the liquids as the counterparts are neglected^43^. In most cases, researchers simply used pure liquids like water (for superhydrophobic surfaces) and *n*-hexadecane (for superamphiphobic surfaces) as the probes. Obviously, the liquid repellency of super anti-wetting coatings is highly dependent on properties of the liquids^25,44–46^. For anti-icing, the interaction between superhydrophobic surfaces and super-cooled water is very different from that at room temperature because of high viscosity of super-cooled water^25,47^. The viscosity of water at −30 °C is ca. 5 times higher than that at room conditions. For superamphiphobic surfaces, pure liquids with different surface tensions ranging from 72.8 mN m^−1^ (water) to 22.5 mN m^−1^ (methanol) were studied.

However, liquids of diverse compositions, e.g., solutions, suspensions and emulsions, are used in academic research, industrial production and our daily life. The properties of these liquids including viscosity, surface tension, density and flow form are far different from those of the frequently used pure liquids in the field of super anti-wetting surfaces. Consequently, the solid-liquid interaction and liquid repellency will be changed evidently, which requires design of novel surface microstructures. Nevertheless, the research about super anti-wetting coatings repelling complex liquids, e.g., high viscosity, high solid content and low surface tension, is very rare. Brown and Bhushan reported superoleophobic polypropylene surfaces exhibiting some repellency towards shampoos^48^. The mixtures of glycerol and water were also used as the model liquids of different viscosity ranging from 0.959 mPa·s (water) to 950 mPa·s (glycerol)^44,49^. Research about super anti-wetting coatings repelling complex liquids is of great importance for their development and applications.

Here, we report preparation of superamphiphobic surfaces with high repellency towards liquids of extremely high viscosity and low surface tension. The surfaces were prepared by constructing a hierarchical micro-/nanostructure on the Cu micropillar arrays followed by modification with perfluorosilane. Hydroxyl-terminated polybutadiene (HTPB) and the HTPB/Al mixture (1:1, w/w) were used as the probing complex liquids with high viscosity, high solid content and low surface tension. HTPB, a viscous mixture rather than a pure compound, is an oligomer of butadiene, and could react with isocyanates to form polyurethane polymers.

## Results

### Properties of probing liquids

**Figure 1:**
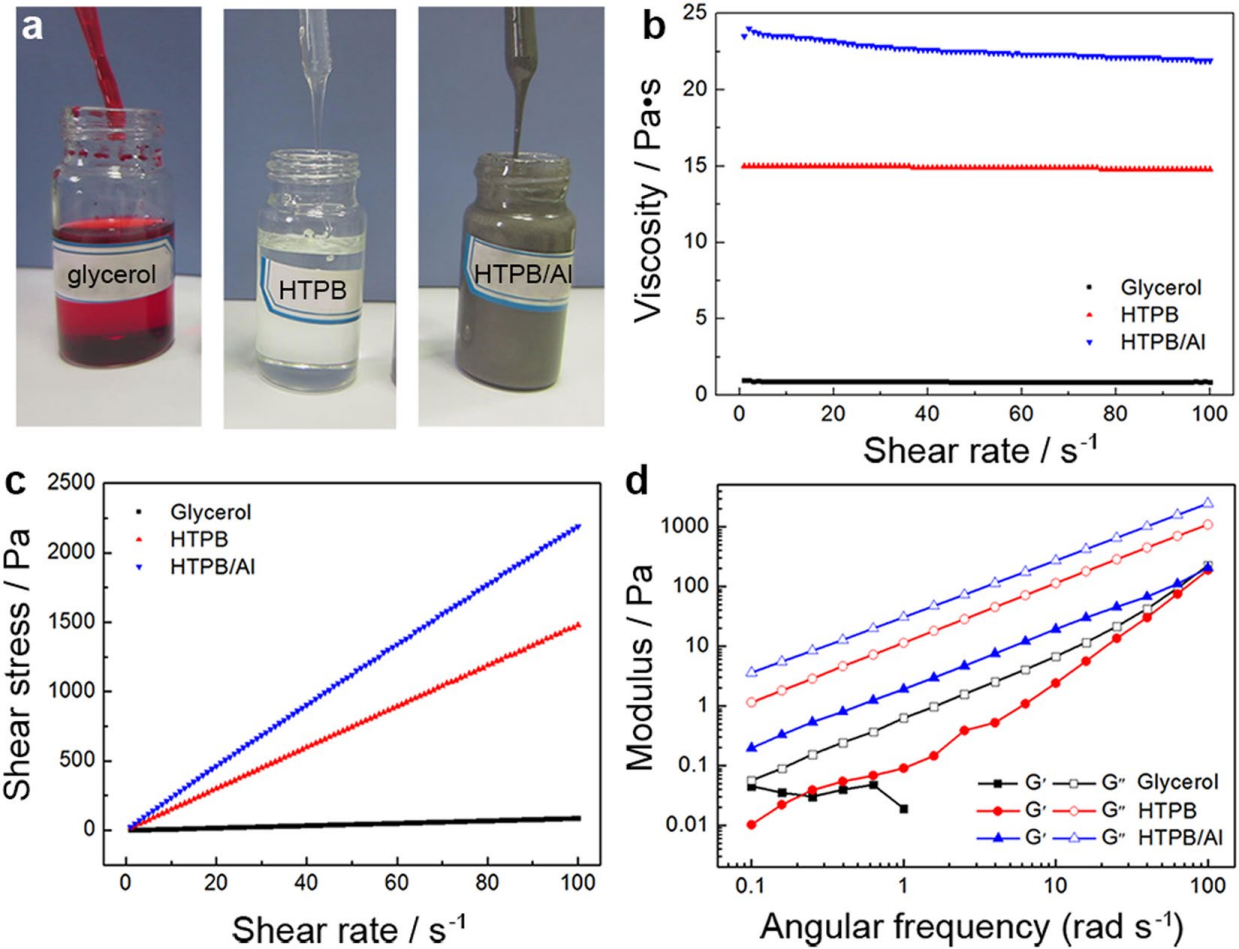
Properties of probing liquids. (**a**) Photographs of glycerol, HTPB and HTPB/Al. (**b**,**c**) Steady and (**c**) dynamic rheological behavior of glycerol, HTPB and HTPB/Al.

HTPB and the HTPB/Al mixture (1:1) were used as the probing complex liquids with high viscosity, high solid content and low surface tension (Fig. 1a). Water and glycerol were also studied for comparison. The dynamic viscosity, surface tension and density of these liquids are listed in Table 1. The dynamic viscosity of HTPB and HTPB/Al is 15 and 24 Pa·s, respectively, which is much higher than that of glycerol, frequently used as the representative viscous liquid. For the injection of HTPB and HTPB/Al, the diameter of the needle must be big enough (outside diameter = 1.65 mm, inside diameter = 1.25 mm) because of their very high viscosity. Also, smaller drops released from the needle with a big diameter cannot be dropped onto the superamphiphobic surfaces because the liquid/needle adhesion force is much large than that between the surfaces and the liquids. Consequently, liquid drops of 20 µL were used for the measurement of contact angles (CAs) and sliding angles (SAs). In addition, the viscosity of the liquids did not show obvious change with increasing the shear rate while the shear stress increased linearly (Fig. 1b–c), indicating that these liquids are the Newtonian fluids^50^. Oscillatory measurements showed that the storage modulus (G’) of the liquids is lower than the loss modulus (G”) in the angular frequency of 0.1–100 rad s^−1^ (Fig. 1d), indicating a viscous response of the liquids^50^. Also, the surface tension of HTPB and HTPB/Al is 38.4 and 42.8 mN m^−1^, respectively, which is much lower than that of water and glycerol.

**Table 1:** Properties of probing liquids.

Liquids	Dynamic viscosity/(Pa·s, 20 °C)	Surface tension/(mN m^−1^, 20 °C)	Density/(g mL^−1^)
Water	0.001	72.4	1.0
Glycerol	0.9	64.1	1.28
HTPB	15	38.4	0.89
HTPB/Al (1:1)	24	42.8	1.02

Dynamic viscosity, surface tension and density of the liquids employed in the experiments.

### Preparation of superamphiphobic Cu/CuO@PFDTCS micropillar arrays

**Figure 2:**
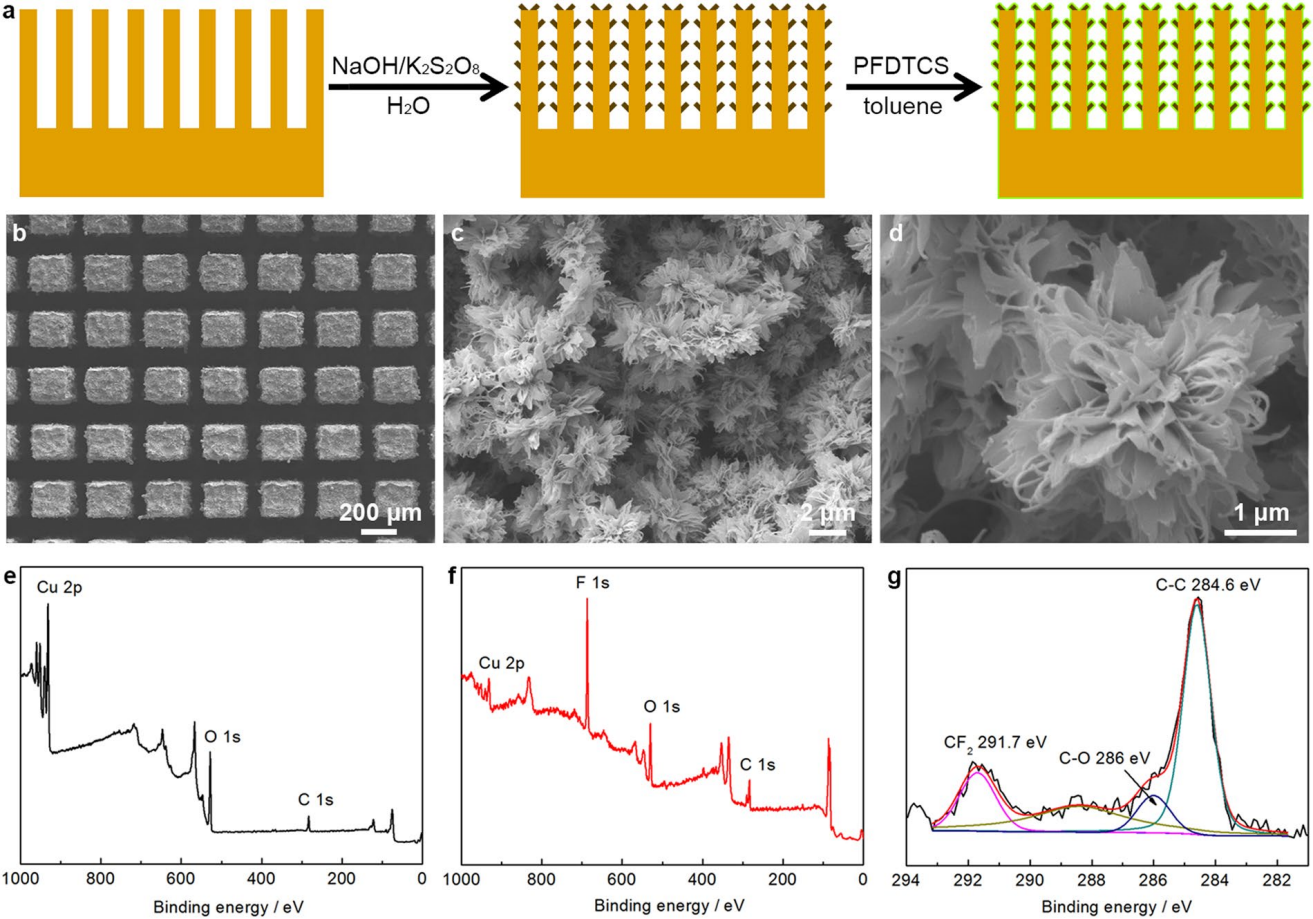
Preparation of superamphiphobic surfaces. (**a**) Schematic illustration of preparation of the superamphiphobic Cu/CuO@PFDTCS micropillar arrays and (**b**–**d**) their SEM images (*D*_pillar_ = 100 μm). XPS spectra of (**e**) Cu micropillar arrays, (**f**) Cu/CuO@PFDTCS micropillar arrays and (**g**) high-resolution C 1s spectrum of Cu/CuO@PFDTCS micropillar arrays.

**Figure 3:**
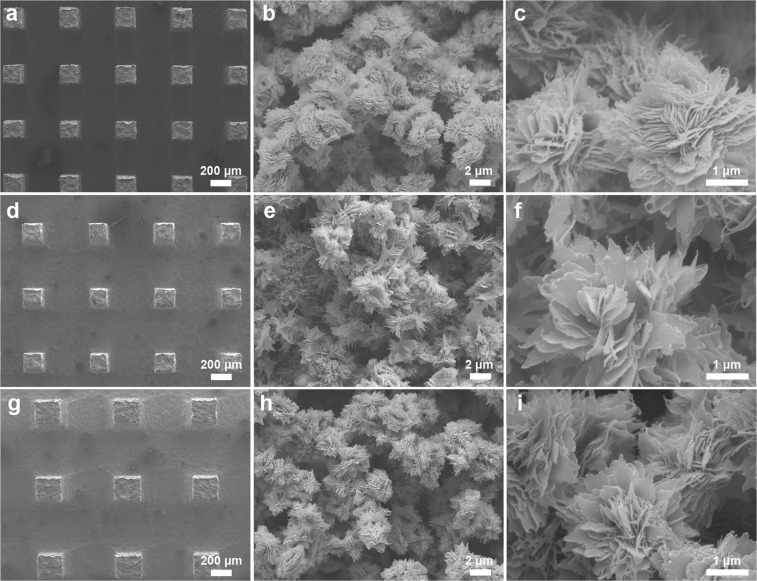
SEM images of Cu/CuO@PFDTCS micropillar arrays with different *D*_pillar_. (**a**–**c**) 300 μm, (**d**–**f**) 400 μm and (**g**–**i**) 500 μm.

We prepared the superamphiphobic Cu/CuO@PFDTCS micropillar arrays using the Cu micropillar arrays with the same pillar size and different pillar distance (*D*_pillar_ = 100, 300, 400 and 500 μm, Supplementary Fig. S1). Firstly, the oxides on the surface of the Cu micropillar arrays were removed by immersion in 0.1 M HCl aqueous solution. Then, the Cu micropillar arrays with metallic gloss were oxidized in an aqueous solution containing NaOH and ammonium persulfate. In this step, the nanostructured CuO layer was formed on the Cu micropillar arrays, and the micro-/nanostructured Cu/CuO micropillar arrays were obtained (Fig. 2a). Finally, the Cu/CuO micropillar arrays were immersed in dry toluene containing *1H*,*1H*,*2H*,*2H*-perfluorodecyltrichlorosilane (PFDTCS). The PFDTCS molecules preferentially anchored onto the hydroxyl groups on the CuO surface^51^, and the superamphiphobic Cu/CuO@PFDTCS micropillar arrays were obtained. The surface of the Cu/CuO@PFDTCS micropillar arrays (Fig. 2b) is very rough compared with the Cu micropillar arrays. This is because CuO nano-flowers resembling miniature replicas of chrysanthemums were formed on the surface of the Cu micropillar arrays (Fig. 2c,d). Modification with PFDTCS did not cause any obvious change of surface morphology^51^. Figure 3 shows the scanning electron microscopy (SEM) images of the Cu/CuO@PFDTCS micropillar arrays with larger *D*_pillar_. There is no difference in the surface morphology of the samples at both low and high magnifications except for the *D*_pillar_. This means the method is reproducible, and the difference in wettability of the samples is only attributed to the *D*_pillar_.

The surface chemical composition of the Cu/CuO@PFDTCS micropillar arrays was studied by X-ray photoelectron spectroscopy (XPS) with the Cu micropillar arrays for comparison. The C 1s, O 1 s and Cu 2p peaks were detected in the XPS spectrum of the Cu micropillar arrays (Fig. 2e). Differently, a very strong F 1s peak appeared in the XPS spectrum, and the CF_2_ peak appeared in the high-resolution C 1s spectrum of the Cu/CuO@PFDTCS micropillar arrays (Fig. 2f,g), which indicate successful bonding of the PFDTCS molecules onto the CuO nano-flowers. Also, the Cu 2p peak became weak (Fig. 2f) owing to deposition of the polymerized PFDTCS layer.

### Superamphiphobicity of Cu/CuO@PFDTCS micropillar arrays

**Figure 4:**
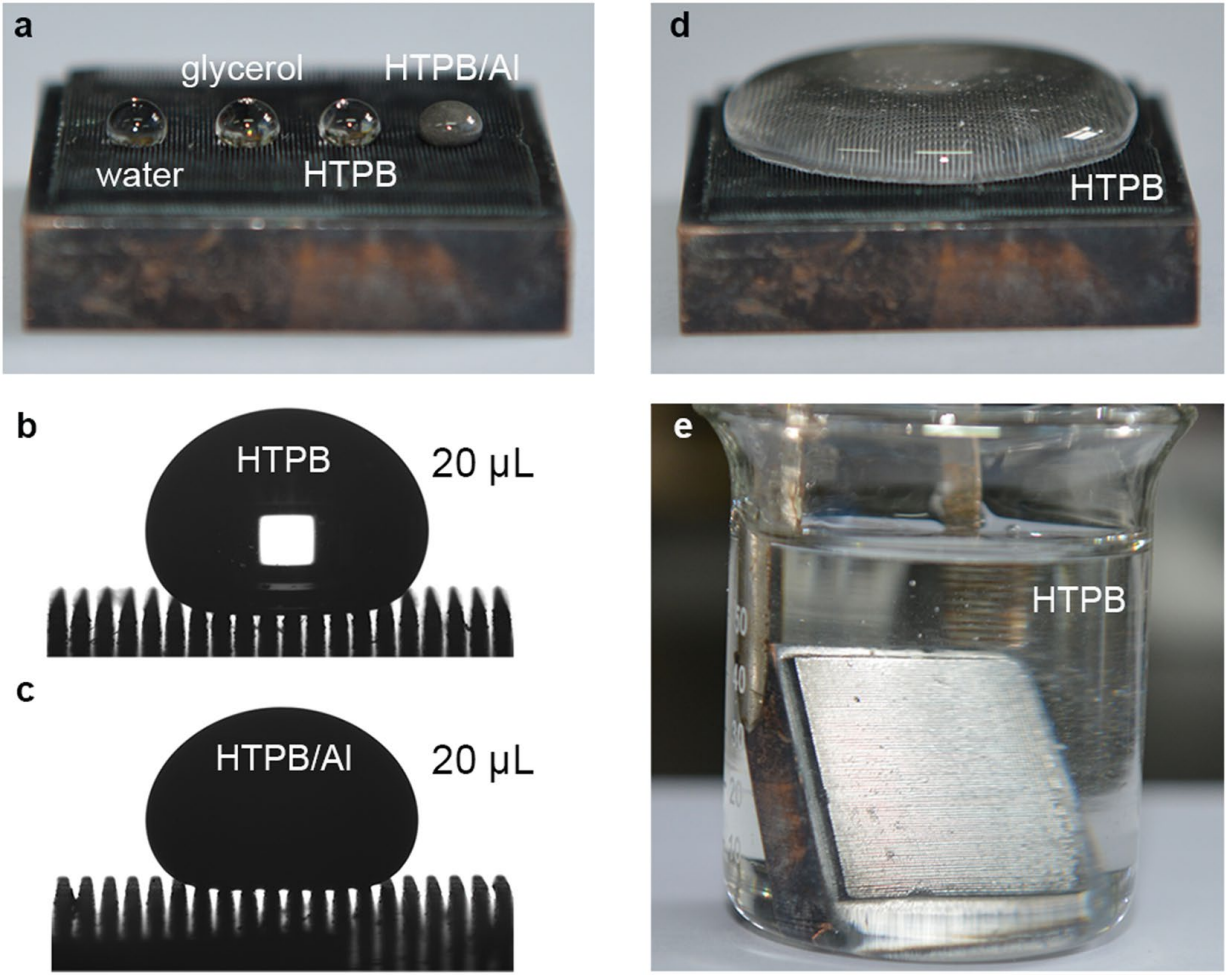
Photographs of the Cu/CuO@PFDTCS micropillar arrays. (**a**–**d**) with various drops and (**e**) immersed in HTPB. *D*_pillar_ = 100 μm.

All the four Cu/CuO@PFDTCS micropillar arrays with different *D*_pillar_ are superamphiphobic owing to the combination of the micro-/nanostructured surface and the abundant perfluorodecyl groups. The CA and SA of various typical liquids, HTPB and HTPB/Al with different surface tensions on the surface of the arrays with a *D*_pillar_ of 100 μm are shown in Table 2. For all of the typical liquids investigated, even 1% cetyltrimethylammonium bromide (CTAB) aqueous solution and *n*-tetradecane, high CA (>157°) and low SA (<10°) on the surface were recorded. For, silicone oil, the SA is ca. 26.1° owing to the very low surface tension. Different from the typical liquids, the HTPB and HTPB/Al drops have smaller CA. In addition, water, glycerol, HTPB and HTPB/Al drops were spherical in shape on the surface of the arrays with a *D*_pillar_ of 100 μm (Fig. 4a). The HTPB and HTPB/Al drops sat very well on the arrays even after being dropped onto the arrays for 10 min (Fig. 4b,c). A larger volume of HTPB formed a liquid pancake on the surface of the arrays without penetrating into the arrays (Fig. 4d). Once immersed in HTPB, a silver mirror phenomenon was observed owing to the existence of a layer of air at the solid-liquid interface (Fig. 4e)^51^. The arrays remained completely dry after taken out of HTPB (Supplementary Movie S1). These phenomena indicate that all the four liquids are in the Cassie-Baxter state on the surface of the Cu/CuO@PFDTCS micropillar arrays with a *D*_pillar_ of 100 μm.

**Table 2:** Superamphiphobicity.

Liquids	CA/°	SA/°	Surface tension/(mN m^−1^, 20 °C)
water	166.7	1.7	72.4
glycerol	162.9	3.7	64.1
diiodomethane	160.2	2.7	44.4
1% CTAB_(aq)_	165.1	2.5	34.9
*n*-hexadecane	160.4	8.3	27.4
*n*-tetradecane	157.1	9.8	25.2
silicone oil	152.7	26.1	19.9
HTBP	151.3	4.0	38.4
HTPB/Al (1:1)	150.4	8.3	42.8

CA and SA of various liquids on the surface of the Cu/CuO@PFDTCS micropillar arrays with a *D*_pillar_ of 100 μm.

The superamphiphobic Cu/CuO@PFDTCS micropillar arrays also showed good mechanical stability according to the sand abrasion test and the water jetting test (Supplementary Fig. S2). These two methods are frequently used for evaluating the mechanical stability of superhydrophobic and superamphiphobic surfaces^22,30^. For the sand abrasion test, 500 g of sea sand microparticles 100–300 µm in diameter impacted the 45° tilted surface from a height of 50 cm in about 10 min. For the water jetting tests, water jet at 50 KPa scoured the 45° tilted surface from a height of 20 cm for a period of time. The changes in the CA_*n*-hexadecane_ and SA_*n*-hexadecane_ after the test were recorded to evaluate the change in the superamphiphobicity and the mechanical robustness of the surface. Although the CA_*n*-hexadecane_ and SA_*n*-hexadecane_ of changed quickly at the beginning of the tests, the surface remained superamphiphobic after abrasion with 500 g sand microparticles or water jetting for half an hour.

**Figure 5:**
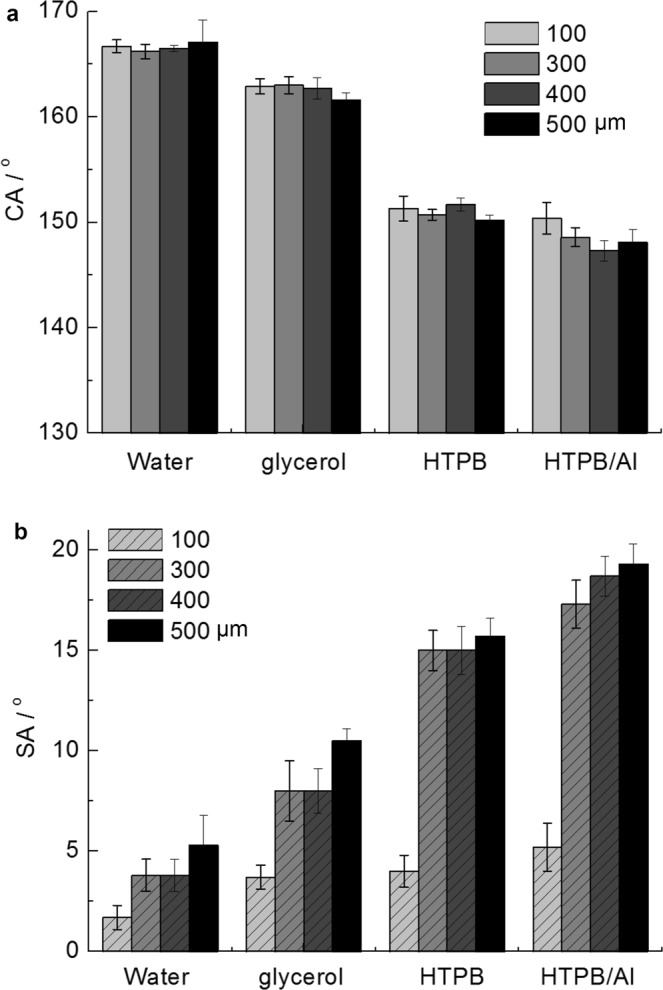
Effect of *D*_pillar_ on superamphiphobicity. (**a**) CAs and (**b**) SAs of water, glycerol, HTPB and HTPB/Al on the surface of the Cu/CuO@PFDTCS micropillar arrays with different *D*_pillar_.

The effect of the *D*_pillar_ on the superamphiphobicity of the Cu/CuO@PFDTCS micropillar arrays is shown in Fig. 5. As can be seen from Fig. 5a, the *D*_pillar_ did not have obvious influence on the CA_water_ (166.2°~167.1°), CA_glycerol_ (161.6°~163.0°) and CA_HTPB_ (150.2°~151.7°). However, the CA_HTPB/Al_ is a bit higher on the arrays with a *D*_pillar_ of 100 μm (150.4°) than those on the other arrays (147.3°~148.6°). On the other hand, the CAs decreased gradually with decreasing the surface tension from 72.4 mN m^−1^ (water) to 38.4 mN m^−1^ (HTPB). This is consistent with all the literatures about superamphiphobic surfaces^6,8^. It should be noted that the CA_HTPB/Al_ is slightly lower than that of the CA_HTPB_ although the surface tension of HTPB/Al (42.8 mN m^−1^) is higher than that of HTPB. This is owing to the very high solid content of HTPB/Al.

Different from the CAs, the *D*_pillar_ had an evident influence on the SAs of the arrays (Fig. 5b), as the SAs are more sensitive than the CAs to the changes in microstructure and surface chemical composition of super anti-wetting surfaces^31^. All the four liquids showed much lower SAs (<6°) on the surface of the arrays with a *D*_pillar_ of 100 μm compared with the arrays with larger *D*_pillar_. The SAs increased with increasing the *D*_pillar_ from 300 to 500 μm, but the difference is small. In addition, on the surface of the arrays with a *D*_pillar_ of 100 μm, the surface tension of the liquids showed small influence on the SAs. All the four liquids showed very low SAs, indicating high superamphiphobicity of the surface. For example, the SA_HTPB_ is 4.0° and the SA_HTPB/Al_ is 5.2°. However, on the arrays with larger *D*_pillar_, water has low SA, but HTPB and HTPB/Al with lower surface tension have much higher SAs. A lower SA means smaller solid-liquid interaction, and is very important for most applications of super anti-wetting surfaces.

**Figure 6:**
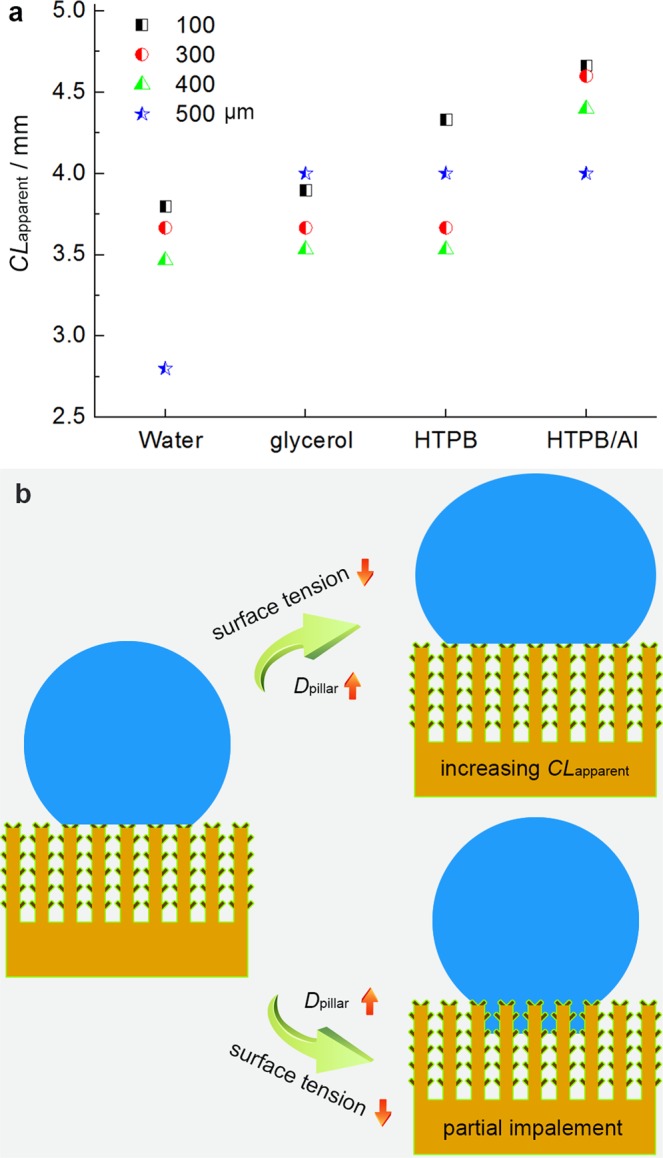
Liquid-solid interaction. (**a**) *CL*_apparent_ of various liquids on the surface of the Cu/CuO@PFDTCS micropillar arrays. (**b**) Schematic illustration of two possible approaches that the Cu/CuO@PFDTCS micropillar arrays support the liquids with changing the *D*_pillar_ and the surface tension.

Besides the superamphiphobic phenomena, CAs and SAs, we also analyzed the solid-liquid apparent contact line (*CL*_apparent_) of various liquids on the surface of the Cu/CuO@PFDTCS micropillar arrays (Fig. 6a and Supplementary Fig. S3). With a *D*_pillar_ of 500 μm, the solid-water *CL*_apparent_ was short, and 3 micropillars were sufficient to support a 20 μL water drop. With decreasing surface tension of the liquid to 64.1 mN m^−1^ (glycerol), the *CL*_apparent_ suddenly increased to 4 mm (4 micropillars) because of the very big *D*_pillar_. With further decreasing the surface tension and increasing the viscosity (HTPB and HTPB/Al), the *CL*_apparent_ remained constant. This is also because of the very big *D*_pillar_, and the HTPB and HTPB/Al drops cannot reach the fifth micropillar. Consequently, the micropillars supported the liquids of lower surface tension by additional contact line at the side of the micropillars, i.e., partial impalement (Fig. 6b). For the other three micropillar arrays, the *CL*_apparent_ is the longest at a *D*_pillar_ of 100 μm followed by a *D*_pillar_ of 300 μm and 400 μm in the case of all the liquids. This means the same drop is supported by more micropillars at smaller *D*_pillar_, efficiently inhibiting impalement into the spaces among the micropillars, i.e. the adhesion force on each pillar is smaller. For example, a 20 μL HTPB was supported by 9 micropillars at a *D*_pillar_ of 100 μm, but was only supported by 4–5 micropillars at a *D*_pillar_ of 300–500 μm. This should be the reason for the aforementioned increasing SAs with increasing the *D*_pillar_. In addition, the micropillar arrays with a *D*_pillar_ of 100 μm supported various liquids very well by continuously increasing the *CL*_apparent_ with decreasing surface tension of the liquids. However, the other arrays with higher *D*_pillar_ of 300 and 400 μm cannot support the liquids well enough, as the *CL*_apparent_ remained constant with decreasing the surface tension, indicating partial impalement. Figure 6b summarized the two possible approaches that the micropillar arrays support the liquids with changing the *D*_pillar_ and the surface tension: i) increasing the *CL*_apparent_, and ii) partial impalement. The Cu/CuO@PFDTCS micropillar arrays with a *D*_pillar_ of 100 μm can efficiently support the liquids of low surface tension without any impalement.

### Kinetic interaction of Cu/CuO@PFDTCS micropillar arrays with various liquids

The kinetic interaction of the Cu/CuO@PFDTCS micropillar arrays with various liquids was studied in this section. The adhesion forces between the Cu/CuO@PFDTCS micropillar arrays and various liquids are shown in Table 3 and Supplementary Fig. S4. The *D*_pillar_ has a great influence on the adhesion forces. The adhesion forces of the liquids on the arrays with a *D*_pillar_ of 100 μm are much lower than those on the arrays with a *D*_pillar_ of 300 μm. Whereas the further increase in the *D*_pillar_ to 500 μm did not have obvious effect on the adhesion forces. For example, the adhesion force of HTPB on the arrays with a *D*_pillar_ of 100 μm is 23.6 μN, whereas the adhesion forces are in the range 69.5–75.5 μN on the arrays with *D*_pillar_ of 300–500 μm. In addition, it was found that the surface tension and viscosity of the liquids also have big influence on the adhesion forces. On all the arrays, the adhesion forces are in the order glycerol < water < HTPB/Al < HTPB, except for small deviation for HTPB and HTPB/Al on the arrays with a *D*_pillar_ of 100 μm. It is a common phenomenon that the adhesion force of glycerol is smaller than that of water regardless of the *D*_pillar_. The difference in surface tension between water and glycerol is small, whereas the viscosity of glycerol is much higher than that of water. So, a higher viscosity is helpful to reduce the adhesion force for liquids of similar surface tension. Compared to water, the viscosity of HTPB is much higher (15 Pa·s *vs* 0.001 Pa·s), which is helpful to reduce the adhesion force. Meanwhile, the surface tension of HTPB is much lower (38.4 mN m^−1^ *vs* 72.4 mN m^−1^), which tend to enhance the adhesion force^47^. Owing to the synergistic effects of viscosity and surface tension, the adhesion forces of HTPB are higher than those of water on the arrays with *D*_pillar_ of 300–500 μm, and the adhesion forces of HTPB and water are almost the same on the arrays with a *D*_pillar_ of 100 μm. This result further proves that a high viscosity of liquids can reduce the adhesion force, and compensate the effect of lower surface tension in increasing the adhesion force. For example, HTPB/Al with higher viscosity and surface tension than HTPB showed obviously lower adhesion forces on the arrays with *D*_pillar_ of 300–500 μm. Thus, the adhesion forces in Table 3 are the end product of *D*_pillar_, viscosity and surface tension. Smaller *D*_pillar_, higher viscosity and higher surface tension contribute to reducing the adhesion force.

**Table 3:** Adhesion forces. Adhesion forces between the Cu/CuO@PFDTCS micropillar arrays and various liquids.

Adhesion forces/μN	*D*_pillar_/μm			
	100	300	400	500
Water	32.4	87.3	86.3	84.3
Glycerol	23.6	75.5	69.5	75.5
HTPB	34.3	116.7	108.9	106.9
HTPB/Al	52.0	91.2	95.2	75.5

The influences of *D*_pillar_, viscosity and surface tension on the adhesion forces were also well evidenced by the kinetic behavior of drops on the Cu/CuO@PFDTCS micropillar arrays. Compared to the arrays with a bigger *D*_pillar_ of 300 μm, the lateral and vertical deformations of the HTPB drops are small on the arrays with a *D*_pillar_ of 100 μm while laterally moving the arrays beneath the drops and lifting the drops vertically from the surface (Supplementary Movies S2 and S3). The smaller the deformation is, the smaller the adhesion force is. In addition, no observable lateral and vertical deformations of the glycerol drop could be detected while moving the arrays (*D*_pillar_ = 100 μm) beneath the drops and lifting the drops vertically from the surface (Supplementary Movies S4 and S5). However, small deformations were detected in the case of water. This difference proved that glycerol with higher viscosity has smaller adhesion force with the arrays compared to water. Furthermore, the smaller deformation of glycerol than HTPB on the arrays (*D*_pillar_ = 100 μm) demonstrated that higher surface tension can reduce the adhesion force (Supplementary Movie S6).

**Figure 7:**
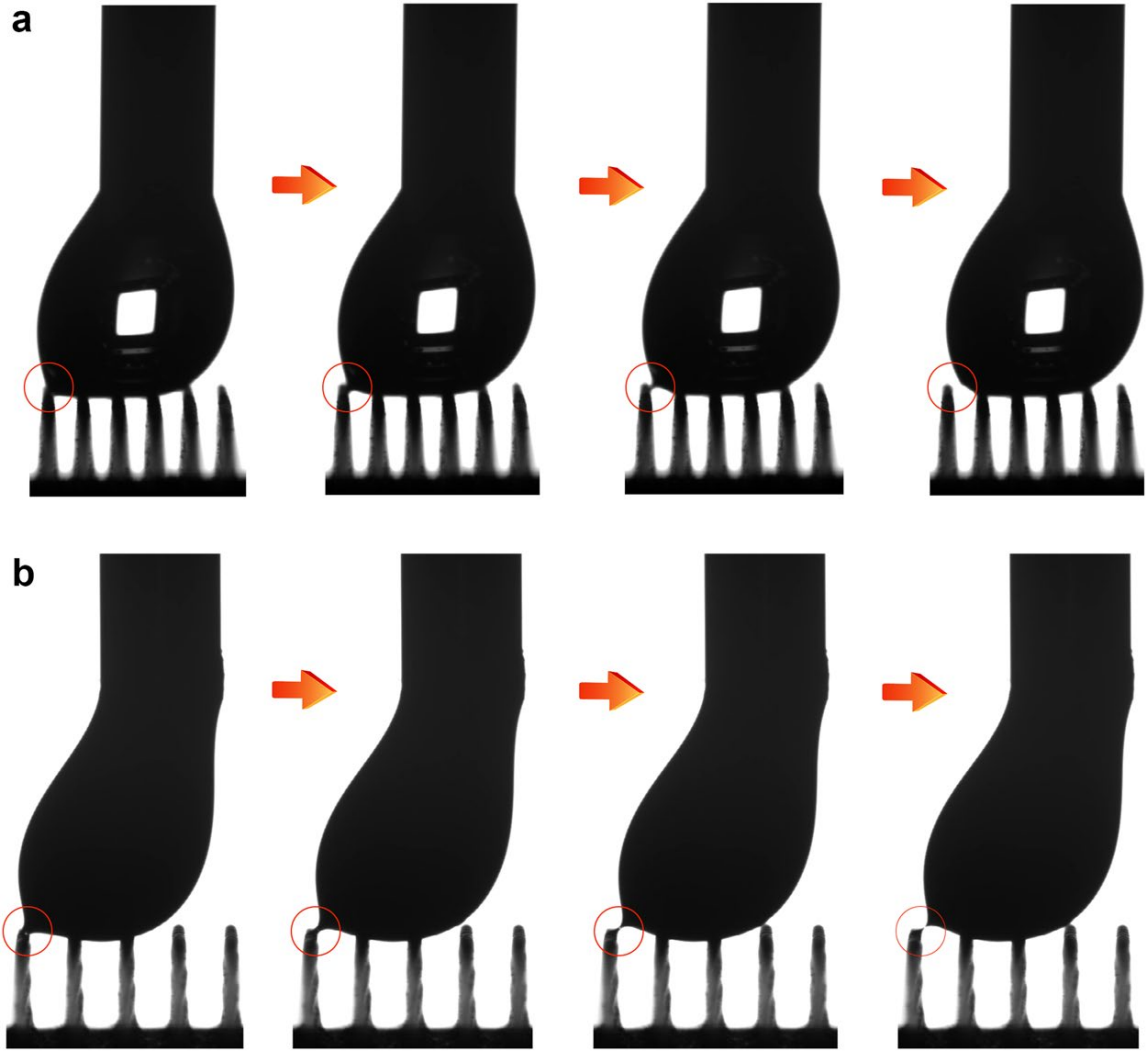
Microcapillary bridge rupture. *In situ* observation of microcapillary bridge rupture on the Cu/CuO@PFDTCS micropillar arrays using (**a**) HTPB (*D*_pillar_ = 300 μm) and (**b**) HTPB/Al (*D*_pillar_ = 500 μm) as the probing liquids (10 μL).

We also studied the kinetic interaction of the Cu/CuO@PFDTCS micropillar arrays with various liquids by checking whether there were residuals on the micropillar arrays after the drop rolled off the surfaces (Table 4). For water and glycerol, no visible liquid “left behind” was observed on all the arrays after the drops rolled off the surfaces. The depinning at the receding edge of the drops was fast and complete. For viscous HTPB, no residual was found on the arrays with a *D*_pillar_ of 100 μm, whereas residuals were detected on the arrays with bigger *D*_pillar_. The residuals of HTPB/Al were apparent on all the arrays. The formation of these residual microdrops arises from microcapillary bridge rupture that occurs during receding events^43^. McCarthy *et al*. verified the conjecture concerning microcapillary bridge rupture using a non-volatile ionic liquid, which was observed via SEM on the staggered rhombus posts of a superhydrophobic surface^43^. We *in situ* observed the process of microcapillary bridge rupture for the first time using highly viscous liquids (HTPB and HTPB/Al) by laterally moving the arrays beneath the drops (Fig. 7 and Supplementary Movie S7). The process of microcapillary bridge rupture is more obvious for HTPB/Al owing to its high viscosity.

**Table 4:** Liquid-solid interaction.

Residuals	*D*_pillar_/μm			
	100	300	400	500
Water	No	No	No	No
Glycerol	No	No	No	No
HTPB	No	Yes	Yes	Yes
HTPB/Al	Yes	Yes	Yes	Yes

Summaries of residuals or not on the Cu/CuO@PFDTCS micropillar arrays after the drops of various liquids rolled off. “No” means no residual, and “Yes” means residual was observed.

### Rolling of various liquids on Cu/CuO@PFDTCS micropillar arrays

Once the gravitational force acting on a sessile liquid drop on a tilted surface overcomes the lateral adhesion force, the drop starts rolling^27^. The rolling velocity of liquid drops on super anti-wetting surfaces is relevant for their diverse applications, such as self-cleaning, anti-icing and microfluidics, etc^27^. Thus, the kinetic interaction of the Cu/CuO@PFDTCS micropillar arrays with various liquids was further studied by recording the rolling velocity of various liquids on the arrays. The motion of liquid drops (20 μL) on the 15° tilted Cu/CuO@PFDTCS micropillar arrays was filmed (Supplementary Fig. S5), and the velocity was calculated as shown in Table 5. In the rolling process, the potential energy of drops was only partially converted to kinetic energy because of energy loss. The energy loss in the course of rolling on solid surfaces arises mainly from viscous dissipation and surface adhesion^47^. Water drops rolled off the arrays (20 mm) very fast, and it is difficult to record the time (~0.2 s) accurately using a normal video camera (25 fps). No obvious influence of the *D*_pillar_ on the velocity was found. The velocity of glycerol is lower than that of water as shown in Supplementary Movie S8 because of it high viscosity, causing viscous dissipation in the rolling process. For glycerol, the contribution of surface adhesion to energy loss should be smaller than that of water, as the adhesion forces are smaller for glycerol on the same arrays. With increasing viscosity and decreasing surface tension of the liquids, the rolling velocity became even lower on all the arrays. For example, the velocity of HTPB is 0.44 mm s^−1^ and the velocity of HTPB/Al is 0.26 mm s^−1^ on the arrays with a *D*_pillar_ of 100 μm. This is owing to the increase of both viscous dissipation (higher viscosity as shown in Table 1) and surface adhesion (higher adhesion force as shown in Table 3). In addition, it was found that the *D*_pillar_ also has big influence on the rolling velocity (Supplementary Movie S9). The arrays with a *D*_pillar_ of 100 μm exhibited the highest velocity among all the arrays for glycerol, HTBP and HTBP/Al. This is attributed to the smallest adhesion forces on the arrays with a *D*_pillar_ of 100 μm. The above results indicate that it is possible to enhance the rolling velocity of viscous liquids by reducing the adhesion force at the solid-liquid interface and by reducing the viscosity of the liquids.

**Table 5:** Average rolling velocity.

Average rolling velocity/(mm s^−1^)	*D*_pillar_/μm			
	100	300	400	500
Water	100	100	100	100
Glycerol	24.39	6.90	5.04	3.66
HTPB	0.44	0.23	0.20	0.19
HTPB/Al	0.26	0.15	0.14	0.11

Average rolling velocity of various liquids (20 μL) on the 15° tilted Cu/CuO@PFDTCS micropillar arrays.

## Discussion

Superamphiphobic surfaces with high repellency towards liquids of extremely high viscosity and low surface tension were prepared. The contact angles, sliding angles, apparent contact line at the solid-liquid interface and adhesion forces of the superamphiphobic surfaces depend on micropillar distance, viscosity and surface tension of the liquids. Smaller micropillar distance, higher viscosity and higher surface tension are helpful to reduce the adhesion force. The process of microcapillary bridge rupture at the solid-liquid interface was *in situ* observed for the first time by using highly viscous liquids. Also, the adhesion forces were reduced, and the rolling velocity of liquids of extremely high viscosity and low surface tension was enhanced by reducing the micropillar distance. The superamphiphobic surfaces may find applications in many fields, especially in the conditions encountering viscous liquids of low surface tension. We believe that the results in this study will shed a light on the design of novel superamphiphobic surfaces with high repellency towards complex liquids. Also, we should pay much attention to the kinetic wettability of super anti-wetting surfaces besides the static wettability.

## Experimental Section

### Materials

The Cu micropillar arrays (2 × 2 cm) with the same pillar size (200 × 200 μm) and different micropillar distance (*D*_pillar_ = 100, 300, 400 and 500 μm) were prepared by Institute of Chemical Materials, China Academy of Engineering Physics. HCl (37%), NaOH, ammonium persulfate, CTAB, toluene, glycerol and ethanol were purchased from China National Medicines Co. Ltd. PFDTCS (97%) was purchased from Gelest. HTPB with an average molecular weight of 3000 was supplied by China Haohua Chemical Group Co., Ltd. Al powder (100 μm) was supplied by Angang Group Aluminium Powder Co., Ltd.

### Preparation of micro-/nanostructured Cu/CuO micropillar arrays

The micro-/nanostructured Cu/CuO micropillar arrays were prepared by modifying a previously reported method^52,53^. First, the Cu micropillar arrays were ultrasonicated for 30 min in water, and then immersed in 0.1 M HCl aqueous solution for 30 s to dissolve the oxides on the surface. The arrays were then rinsed with deionized water, and dried under a nitrogen flow. Subsequently, the Cu micropillar arrays were oxidized in 200 mL of an aqueous solution containing 5 M NaOH and 0.3 M ammonium persulfate at room conditions for 2 h. In this step, the nanostructured CuO layer was grown on the micropillar arrays. The as-prepared Cu/CuO micropillar arrays were rinsed with deionized water, and then dried under a nitrogen flow.

### Preparation of superamphiphobic Cu/CuO@PFDTCS micropillar arrays

The micro-/nanostructured Cu/CuO micropillar arrays were immersed in 150 mL of dry toluene, and then 40 µL of PFDTCS was added. The sample was kept in the above solution for 12 h at room temperature to ensure complete modification of the CuO layer. The as-prepared Cu/CuO@PFDTCS micropillar arrays were washed with 20.0 mL of dry toluene and dried under a nitrogen flow.

### Measurement of wettability

The CAs and SAs of the probing liquids were measured on a Contact Angle System OCA20 (Dataphysics, Germany) equipped with a tilting table. The syringe was positioned in a way that the liquid drops (20 µL) could contact surface of the samples before leaving the needle. It should be noted that the HTPB and HTPB/Al drops of smaller size are difficult to be dropped onto the surfaces because of their very high viscosity. We measured the CA of water and *n*-hexadecane using drops of different volumes (Supplementary Table S1). We found that the drop volume has very small influence on the CA measurements on surface of the superamphiphobic Cu/CuO@PFDTCS micropillar arrays. Tilting angle of the table was adjustable (0°–70°), and the subsequent measurement of the SAs was allowed at the same position on the sample. The rolling velocity was measured using a timekeeper while moving of a liquid drop on the 15° tilted surface. A minimum of six readings were recorded for each sample, and the average values with standard errors were reported.

### Measurement of adhesion forces

The interfacial adhesive forces between the liquids and the surfaces were measured using a Force Tensiometer K100 (Krüss, Germany) at room temperature. The surface was placed on the balance table and moved upward at a constant speed of 3 mm min^−1^ until it contacted the probing liquid drop suspended with a copper ring, and continuously moved upward for 0.5 mm. Then, the surface was moved downward at a constant speed of 1 mm min^−1^ until the sample broke away from the drop.

### Characterization

The micrographs of the samples were taken using SEM (CamScan Apollo300). Before SEM observation, all samples were fixed on Al stubs and coated with gold (~7 nm). The XPS spectra of samples were obtained using a VG ESCALAB 250 Xi spectrometer equipped with a monochromated Al Kα X-ray radiation source and a hemispherical electron analyzer. The spectra were recorded in the constant pass energy mode with a value of 100 eV, and all binding energies were calibrated using the C 1s peak at 284.6 eV as the reference. Viscoelastic properties of the probing liquids were recorded using a rheometer (Anton Paar Physica, MCR102) at 20 °C. The shear rate was set to be increased from 0.1 to 100 s^−1^ during the test. The changes in the shear stresses with the shear rates were recorded by the software of the rheometer. The surface tension of the liquids was measured using a Force Tensiometer K100 (Krüss, Germany) at 20 °C. The density of the liquids was measured using a previously reported method^47^.

## Supplementary information

Supplementary Information

Supplementary Movie S1

Supplementary Movie S2

Supplementary Movie S3

Supplementary Movie S4

Supplementary Movie S5

Supplementary Movie S6

Supplementary Movie S7

Supplementary Movie S8

Supplementary Movie S9
