# Supplementary material for: Superamphiphobic Cu/CuO Micropillar Arrays with High Repellency Towards Liquids of Extremely High Viscosity and Low Surface Tension
Source: Sci Rep. 2019 Jan 24;9:702. doi: 10.1038/s41598-018-37368-y (PMC6346034; doi:10.1038/s41598-018-37368-y)
Supplement: Supplementary file 1 — Supplementary Information [file 41598_2018_37368_MOESM1_ESM.pdf]

Supporting information

Superamphiphobic Cu/CuO Micropillar Arrays with High Repellency Towards Liquids of Extremely High Viscosity and Low Surface Tension

Qing Zhu,[1†] Bucheng Li,[2†] Shangbin Li[1]* Guan Luo,[1] Baohui Zheng,[1] & Junping Zhang[2]*

[1]Institute of Chemical Materials, China Academy of Engineering Physics, 621999, Mianyang, P. R. China

[2]Key Laboratory of Clay Mineral Applied Research of Gansu Province, Lanzhou Institute of Chemical Physics, Chinese Academy of Sciences, 730000, Lanzhou, P. R. China

[†]These authors contributed equally to this work.

*Correspondence to caeplisb@163.com, jpzhang@licp.cas.cn

Drop volume / µL	5	10	20
CA_{Water}	167.3°	166.9°	166.7°
$CA_{n\text{-hexadecane}}$	161.2°	160.8°	160.4°

Supplementary Table S1. CA of water and *n*-hexadecane measured using drops of different volumes.

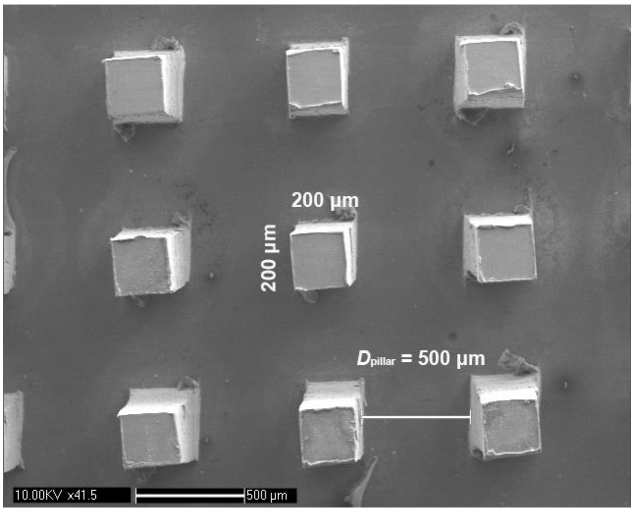

Supplementary Fig. S1. SEM image of the Cu micropillar arrays (D_{pillar} = 500 µm).

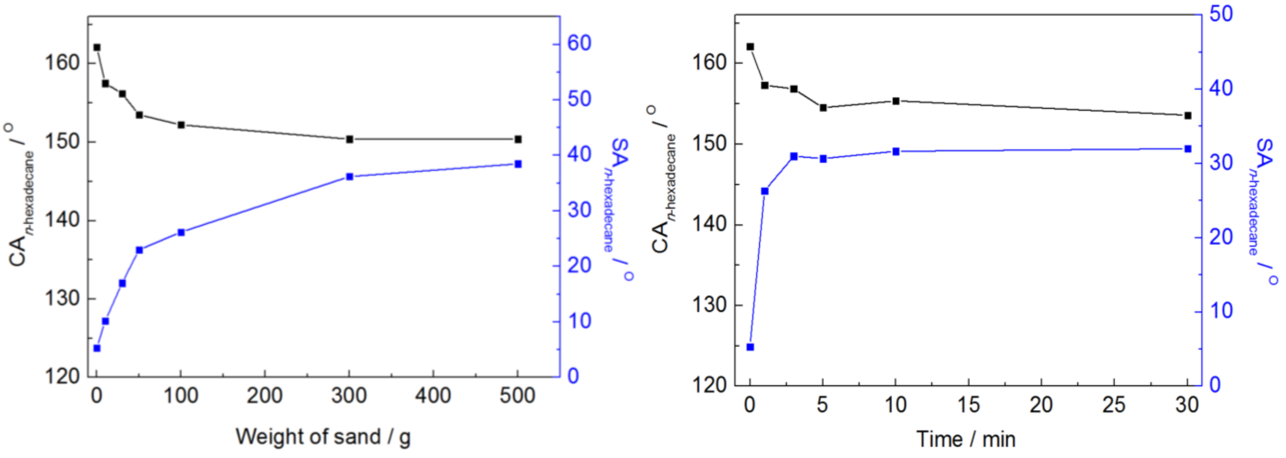

(a)

(b)

Supplementary Fig. S2. Variation of $CA_{n\text{-hexadecane}}$ and $SA_{n\text{-hexadecane}}$ with (a) weight of sand in the sand abrasion test and (b) water jetting time in the water jetting test.

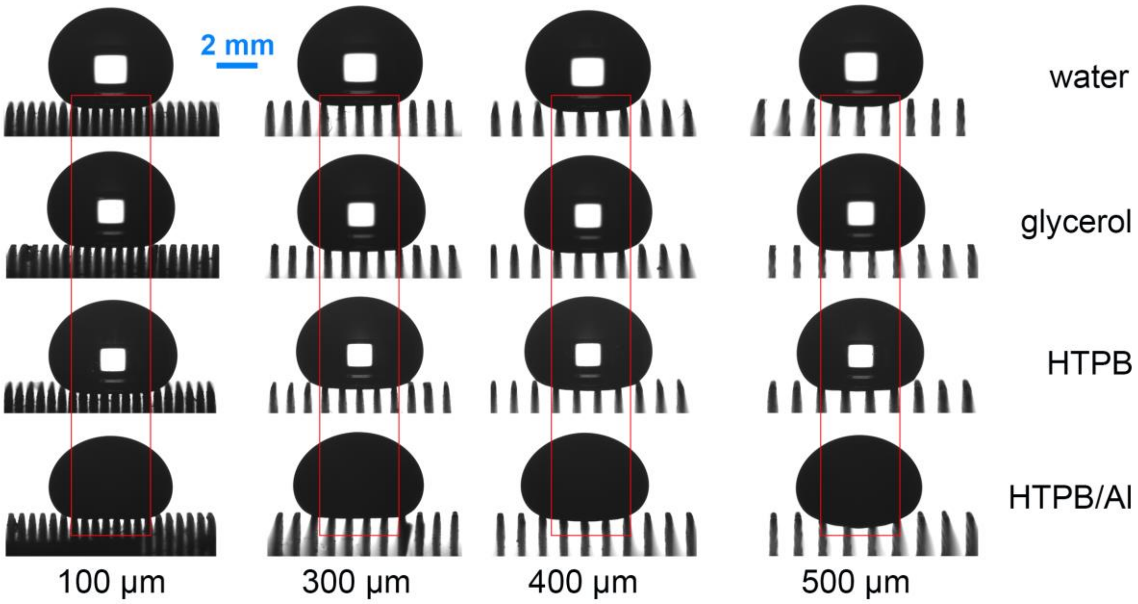

Supplementary Fig. S3. Solid-liquid contact line of various liquids on the surface of the Cu/CuO@PFDTCS micropillar arrays.

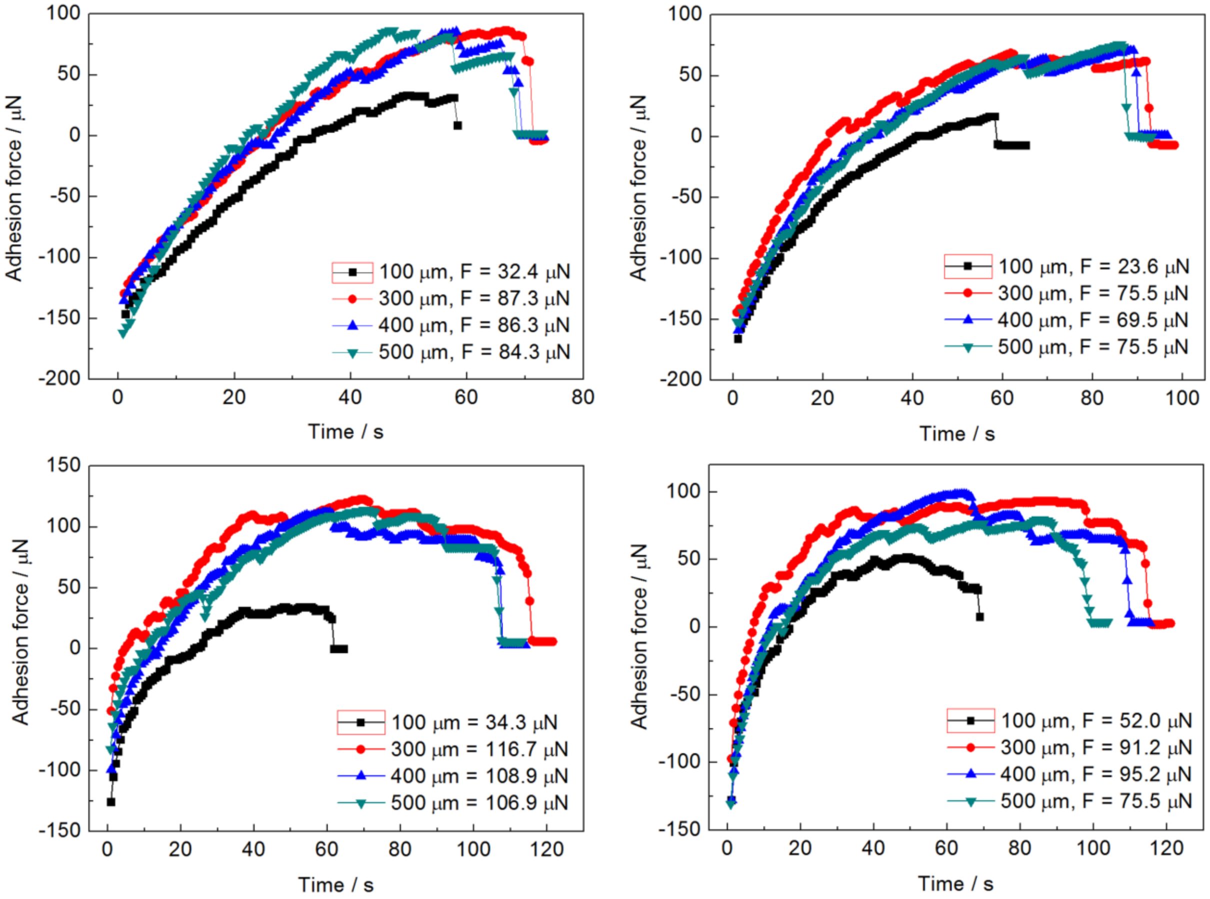

Supplementary Fig. S4. Adhesion forces between the Cu/CuO@PFDTCS micropillar arrays and (a) water, (b) glycerol, (c) HTPB and (d) HTPB/Al.

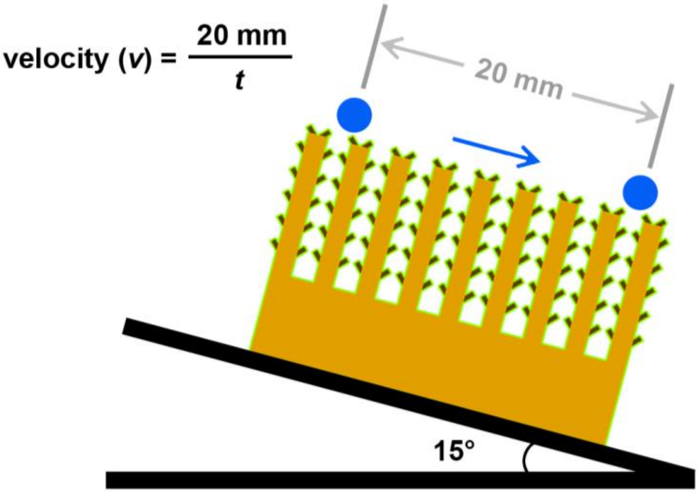

Supplementary Fig. S5. Schematic illustration for measurement of the rolling velocity on the Cu/CuO@PFDTCS micropillar arrays.

Supplementary Movie S1. Immersing the Cu/CuO@PFDTCS micropillar arrays (D_{pillar} = 100 μm) in HTPB and then taking out of HTPB.

Supplementary Movie S2. Horizontal deformation of the HTPB drop on the Cu/CuO@PFDTCS micropillar arrays with different D_{pillar} (100 μm *vs* 300 μm) while horizontally moving the arrays beneath the drop.

Supplementary Movie S3. Vertical deformation of the HTPB drop on the Cu/CuO@PFDTCS micropillar arrays with different D_{pillar} (100 μm *vs* 300 μm) while lifting the drop vertically from the surface.

Supplementary Movie S4. Horizontal deformation of the glycerol and water drops on the Cu/CuO@PFDTCS micropillar arrays (D_{pillar} = 100 μm) while horizontally moving the arrays beneath the drops.

Supplementary Movie S5. Vertical deformation of the glycerol and water drops on the Cu/CuO@PFDTCS micropillar arrays (D_{pillar} = 100 μm) while lifting the drops vertically from the surface.

Supplementary Movie S6. Vertical deformation of the glycerol and HTPB drops on the Cu/CuO@PFDTCS micropillar arrays (D_{pillar} = 100 μm) while lifting the drops vertically from the surface.

Supplementary Movie S7. *In situ* observation of microcapillary bridge rupture on the Cu/CuO@PFDTCS micropillar arrays (D_{pillar} = 500 μm) using HTPB/Al as the probing liquid.

Supplementary Movie S8. Rolling velocity of various liquids (20 μL) on the 15° tilted Cu/CuO@PFDTCS micropillar arrays (D_{pillar} = 100 μm).

Supplementary Movie S9. Rolling velocity of glycerol (20 μL) on the 15° tilted Cu/CuO@PFDTCS micropillar arrays with different D_{pillar}.
